# Recurrent Spontaneous Pneumothorax as a Manifestation of the Birt-Hogg-Dubé Syndrome: A Case Report

**DOI:** 10.7759/cureus.84890

**Published:** 2025-05-27

**Authors:** Farman h Fatah, John T Watson

**Affiliations:** 1 General Medicine, College of Medicine, University of Sulaymaniyah, Sulaymaniyah, IRQ; 2 Pulmonology and Critical Care, Sentara Martha Jefferson Hospital, Charlottsville, USA

**Keywords:** asthma, bhd syndrome, fcln gene mutation, pulmonary cyst, recurrent spontaneous pneumothorax

## Abstract

Birt-Hogg-Dubé syndrome (BHDS) is a rare autosomal dominant disorder caused by mutations in the folliculin (*FLCN*) gene, characterized by a clinical triad of pulmonary cysts with spontaneous pneumothorax, cutaneous fibrofolliculomas, and renal tumors. Pulmonary manifestations may occur in isolation, making early diagnosis challenging. This report describes the case of a 36-year-old woman with a history of right-sided spontaneous pneumothorax, managed with video-assisted thoracoscopic surgery (VATS) and pleurodesis, who re-presented seven years later with a left-sided pneumothorax. Conservative management failed, necessitating VATS pleurodesis and wedge resection of a ruptured subpleural bleb. High-resolution chest imaging revealed bilateral thin-walled pulmonary cysts, and subsequent genetic testing confirmed a pathogenic FLCN mutation, establishing the diagnosis of BHDS. This case underscores the importance of considering BHDS in patients with recurrent spontaneous pneumothorax, particularly in non-smokers and those without underlying lung disease. The recurrence of pneumothorax at a relatively early age highlights the natural course of BHDS and the tendency for pulmonary cyst rupture. It also reinforces the value of early genetic testing and a high index of suspicion for prompt diagnosis. Management extends beyond acute care, encompassing long-term surveillance for renal tumors, pulmonary function monitoring, and family genetic counseling. Recognition of atypical presentations without cutaneous or renal involvement is crucial, as pulmonary symptoms may be the sole initial manifestation. This report contributes to growing awareness of BHDS and emphasizes a multidisciplinary approach for optimal patient outcomes.

## Introduction

Birt-Hogg-Dubé syndrome (BHDS) is a rare autosomal dominant genodermatosis caused by mutations in the folliculin (*FLCN*) gene on chromosome 17p11.2 [1]. First described in 1977, BHDS is characterized by a clinical triad of pulmonary cysts with spontaneous or recurrent pneumothorax, benign skin tumors known as fibrofolliculomas, and a markedly increased lifetime risk of renal neoplasia [2]. The *FLCN* gene encodes the folliculin protein, a tumor suppressor involved in key cellular signaling pathways, including the mTOR pathway, which plays a role in cellular growth, differentiation, and metabolism [3]. FLCN protein is expressed in multiple organs, particularly the lungs, kidneys, and skin [4].

Although the estimated prevalence of BHDS is about two cases per million, its actual incidence is believed to be higher due to frequent underdiagnosis and highly variable clinical presentation, even among members of the same family [5]. While cutaneous lesions often prompt initial suspicion, pulmonary symptoms such as spontaneous pneumothorax may be the first or only manifestation, particularly in younger patients without a history of smoking or underlying lung disease [6,7]. Pulmonary cysts are present in approximately 67-90% of BHDS patients, typically in the basal regions of the lungs [8]. Fibrofolliculomas, the hallmark skin lesions, occur in 58-90% of cases, and renal tumors are seen in about 30% of patients, with a notably increased risk for renal cell carcinoma in individuals over 70 years of age [9]. 

The diagnosis of BHDS is often suspected in patients presenting with cystic lung lesions in the context of a family history of related manifestations, recurrent pneumothorax, and characteristic dermatologic findings. Definitive diagnosis is established through genetic testing confirming pathogenic variants in the* FLCN* gene. Differential diagnoses for cystic lung lesions include Langerhans cell histiocytosis, lymphangioleiomyomatosis (LAM), and other conditions associated with a high risk of secondary spontaneous pneumothorax, which must be carefully distinguished from BHDS.

Here, we describe the case of a 36-year-old woman with a history of spontaneous right-sided pneumothorax who presented again seven years later with left-sided pneumothorax. This case emphasizes the importance of considering BHDS in patients with recurrent pneumothorax and the critical role of genetic testing and long-term surveillance in preventing potentially life-threatening complications such as renal malignancy.

## Case presentation

A 36-year-old woman presented to the emergency department with acute left-sided chest pain and shortness of breath. She had a history of asthma and a prior right-sided spontaneous pneumothorax seven years earlier, which was treated with video-assisted thoracoscopic surgery (VATS) and pleurodesis. Her family history was notable for asthma on her mother’s side and colonic cancer on her father’s side. She denied any history of smoking or environmental exposures.

**Figure 1:**
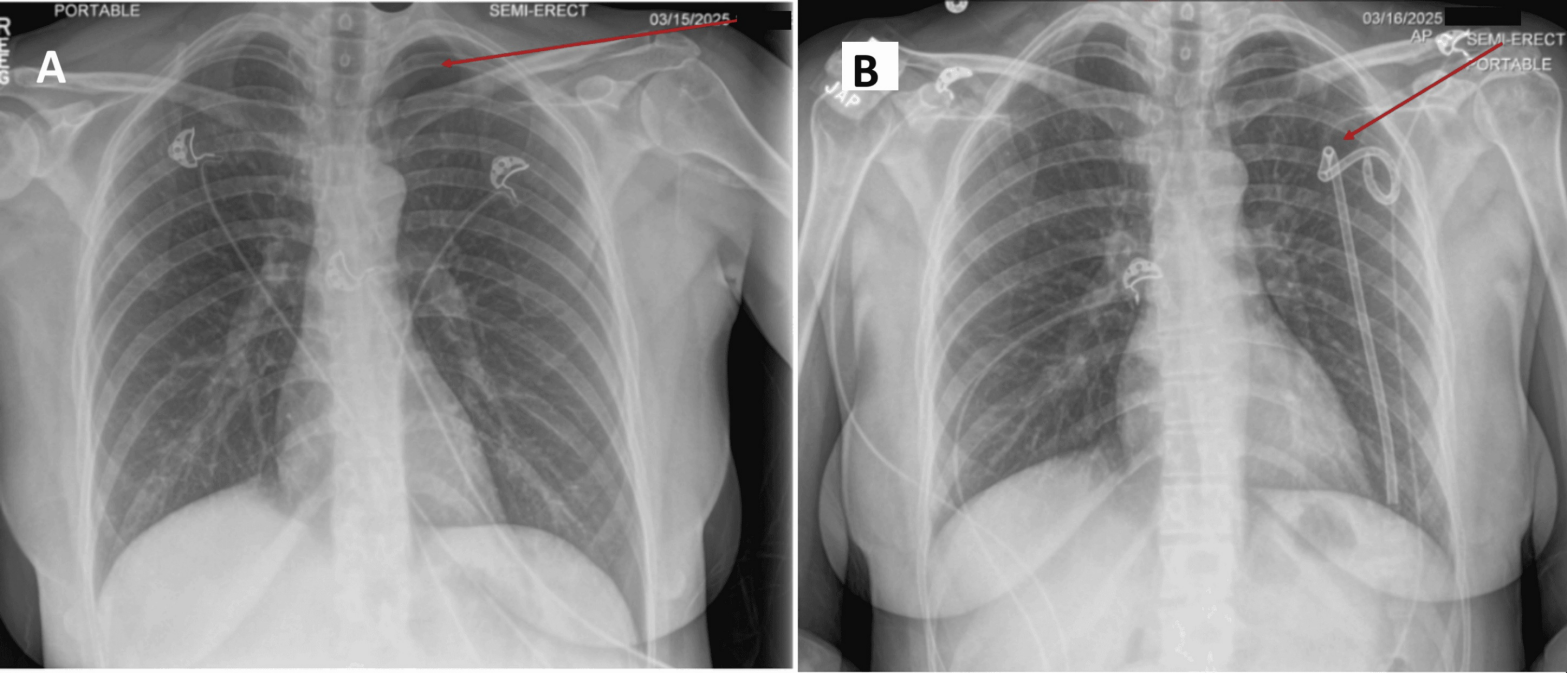
Portable chest X-ray showing (A) a left apical pneumothorax with decreased haziness on the left, and (B) the pigtail catheter that was inserted to drain the intrapleural air.

On physical examination, breath sounds were markedly decreased over the left hemithorax. A chest X-ray was performed, revealing a left-sided apical pneumothorax characterized by a region of radiolucency with absent lung markings, indicating air accumulation in the pleural space (Figure 1A). A pigtail catheter was inserted to relieve the pneumothorax (Figure 1B).

Despite appropriate pigtail placement and conservative management, the pneumothorax persisted. She was referred for surgical evaluation and underwent VATS with pleurodesis and wedge resection. Intraoperatively, a ruptured subpleural bleb in the left upper lobe was identified and excised. Histopathology of the resected tissue was non-specific and showed no malignancy.

**Figure 2:**
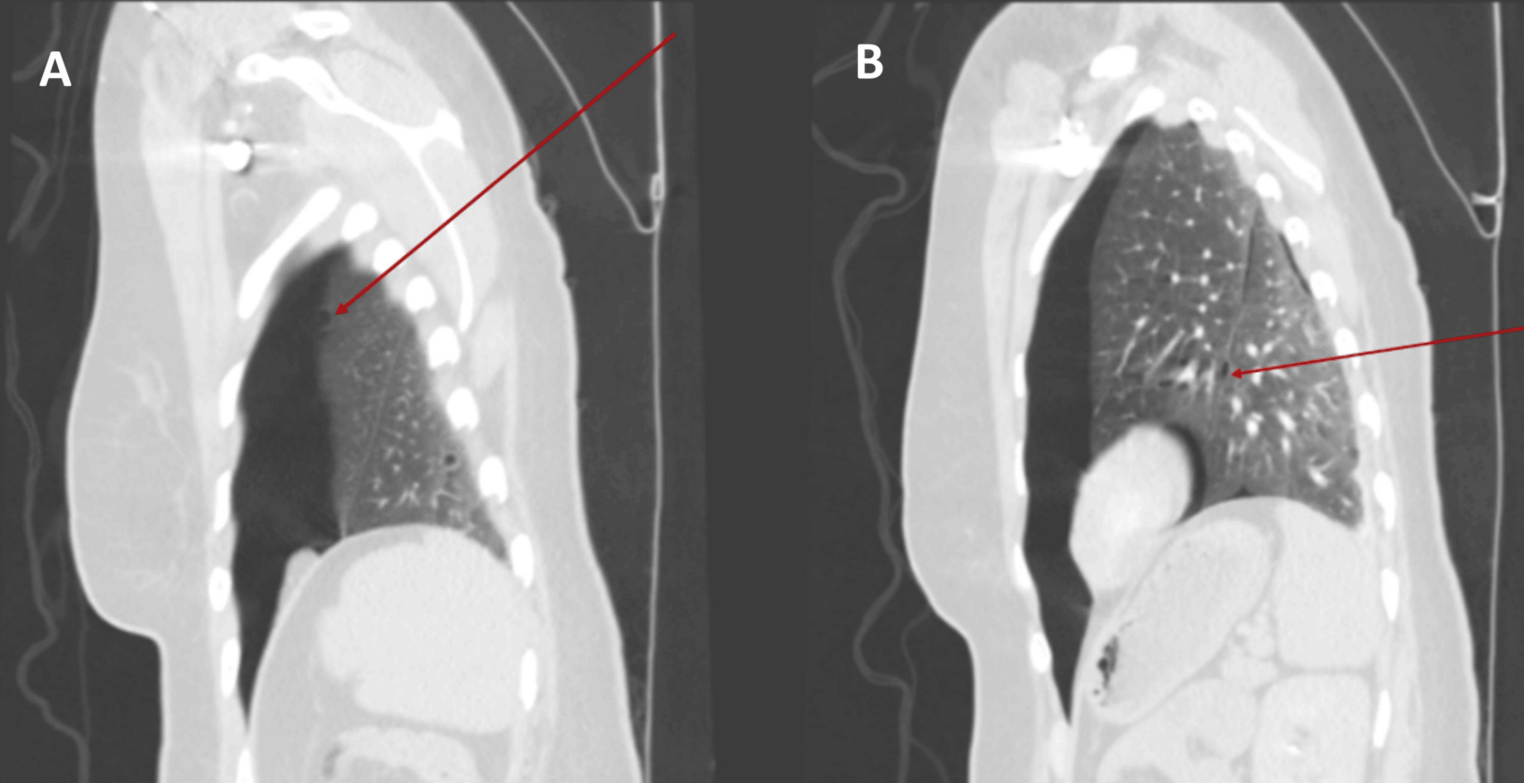
High-resolution CT chest (sagittal view) showing (A) the site of bleb rupture in the left upper lobe, and (B) additional thin-walled cystic lesions within the pulmonary parenchyma.

**Figure 3:**
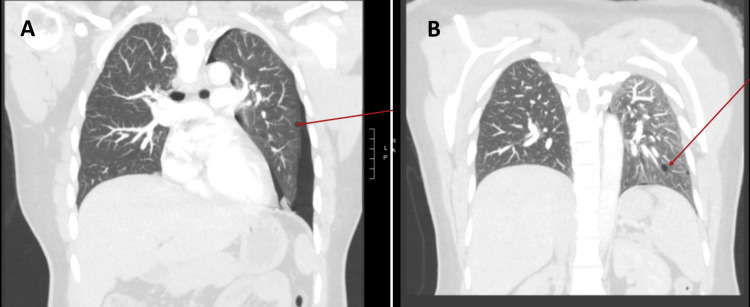
High-resolution CT chest (coronal view) showing (A) a clear view of the left upper lobe bleb rupture site, and (B) scattered cystic lesions.

**Figure 4:**
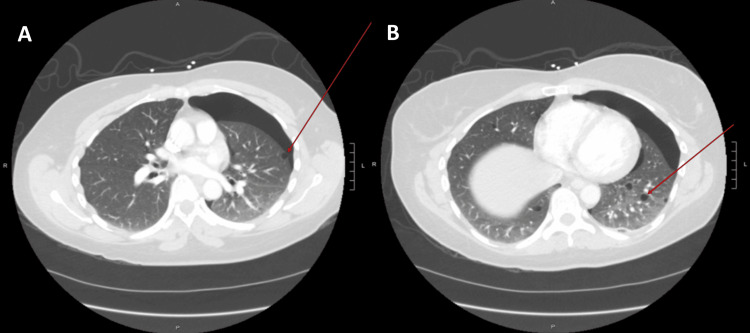
High-resolution CT chest (axial view) showing (A) the ruptured bleb in the left upper lobe, and (B) multiple bilateral cystic lesions in the lung parenchyma.

High-resolution computed tomography (HRCT) of the chest was performed to investigate the underlying cause. Imaging revealed the site of bleb rupture and multiple thin-walled cysts scattered in the lung parenchyma. These abnormalities were well-visualized in the sagittal view (Figure 2), coronal view (Figure 3), and axial view (Figure 4), with the cystic lesions distributed bilaterally, raising suspicion for an underlying cystic lung disease.

Given the history of recurrent spontaneous pneumothorax, the presence of bilateral pulmonary cysts, and a family history of cancer, BHDS was suspected. Genetic testing for a mutation in the *FLCN* gene was performed and confirmed the diagnosis. The test included both sequencing and deletion/duplication analysis of the *FLCN* gene, using transcript reference NM_144997.6. The identified variant was c.1285dup (p.His429Profs*27), a pathogenic duplication that leads to a frameshift and premature protein termination, consistent with a diagnosis of BHDS. A summary of the genetic testing findings is provided in Table 1.

**Table 1:** Summary of genetic testing confirming the diagnosis of Birt-Hogg-Dubé syndrome

Test Name	Gene Analyzed	Transcript Reference	Variant Identified	Interpretation
*FLCN* Sequencing and Deletion/Duplication	FLCN	NM_144997.6	c.1285dup (p.His429Profs*27)	Pathogenic variant consistent with BHDS

The patient had no known renal or dermatologic manifestations at the time of diagnosis. She continued to experience mild postoperative dyspnea and chest discomfort but had resumed daily activities and returned to work. She was scheduled for routine follow-up, including pulmonary function testing and renal surveillance imaging, in accordance with BHDS management guidelines.

## Discussion

Pulmonary manifestations are often the earliest and most prominent clinical feature of Birt-Hogg-Dubé Syndrome (BHDS), frequently preceding skin or renal findings [10]. The syndrome is characterized by multiple bilateral pulmonary cysts, which tend to be irregular, thin-walled, and predominantly located in the basal and subpleural regions of the lungs [6,7]. These cysts predispose affected individuals to spontaneous pneumothorax, which can be the first, and sometimes only, presenting symptom [6].

Our patient experienced two episodes of spontaneous pneumothorax over a span of seven years, with the most recent one requiring surgical intervention after conservative measures failed. Imaging revealed multiple bilateral lung cysts, and the absence of a smoking history or occupational exposures further raised suspicion of a genetic etiology. This was confirmed through molecular testing, which identified a pathogenic mutation in the *FLCN* gene, establishing the diagnosis of BHDS.

One of the key challenges in diagnosing BHDS lies in its variable presentation. While the classical triad includes skin fibrofolliculomas, renal tumors, and pulmonary cysts [11], some individuals, like our patient, may present solely with pulmonary involvement. This phenotypic variability can lead to delayed or missed diagnoses, particularly when cutaneous or renal signs are absent or subtle. approximately 41% of pulmonary cysts can present with spontaneous pneumothorax and with a recurrence rate of 41% [12]. The majority of patients (> 90 %) develop multiple fibrofolliculomas, especially on the face and upper trunk, in the second or third decade of life [13], with dermatologic findings serving as the first clinical clue in 25-50% of cases [14]. Renal tumors are observed in nearly 30% of patients, at a mean age of 50 years [15]. It is now well recognized that pulmonary cysts often appear earlier than other manifestations and may be the only finding for years [7,16]. This underscores the importance of considering BHDS in the differential diagnosis of spontaneous pneumothorax, especially when it is recurrent or associated with atypical cystic lung disease.

The risk of spontaneous pneumothorax in individuals with BHDS is significantly higher than in the general population [17], with a markedly elevated rate of recurrence if the underlying condition is not identified and managed. Recognizing this pattern is critical, as failure to diagnose BHDS can delay important long-term surveillance and preventive care.

Beyond pulmonary complications, BHDS is associated with an increased lifetime risk of developing renal tumors, which are often asymptomatic in the early stages but may become life-threatening if undetected. Therefore, all patients diagnosed with BHDS should undergo routine renal imaging, preferably with MRI, to facilitate early detection [6,16]. RCC symptoms, unfortunately, often appear late and may include hematuria, flank pain, fatigue, and palpable tumors, with advanced cases presenting with bone pain, anemia, or weight loss. As recommended, all BHDS patients should begin abdominal screening at diagnosis and continue regular surveillance even if initial imaging is negative. Preferred imaging modalities include ultrasonography, CT, or MRI. According to current guidelines, BHDS patients without renal tumors should undergo abdominal MRI screening every 36 months, depending on individual risk factors [18]. Those with renal tumors <1 cm should receive annual MRI, while tumors >1 cm warrant more frequent monitoring based on size, location, and growth rate. In our case, the patient was counseled on this risk and advised to begin regular surveillance.

Given the autosomal dominant inheritance of BHDS, genetic counseling and testing should be offered to first-degree relatives [6]. Early identification of family members allows for timely surveillance, particularly for renal complications [15], and the opportunity to intervene before serious manifestations occur.

This case highlights the critical importance of recognizing BHDS in patients presenting with recurrent pneumothorax and bilateral basal lung cysts [19], even in the absence of skin or renal findings. Early diagnosis enables appropriate management, minimizes complications, and allows for family-based genetic risk assessment and screening.

## Conclusions

This case underscores the importance of considering BHDS in patients presenting with recurrent spontaneous pneumothorax, particularly when bilateral pulmonary cysts are evident and no other clear etiology is identified. Surgical intervention, such as VATS pleurodesis, may be necessary when conservative approaches fail. Genetic confirmation through *FLCN* mutation testing is crucial, not only to establish a definitive diagnosis but also to initiate appropriate long-term surveillance for potentially life-threatening renal malignancies.

Early recognition of BHDS allows for timely management, preventive care, and genetic counseling for at-risk family members. Importantly, a multidisciplinary approach involving pulmonologists, thoracic surgeons, radiologists, dermatologists, and urologists is essential for comprehensive assessment, accurate diagnosis, and proactive monitoring. Coordinated care ensures that subtle manifestations are not overlooked and that patients receive tailored interventions to improve outcomes and prevent complications.
